# Chronotypes-personality behavioural syndromes in wild marine fish

**DOI:** 10.1038/s41598-023-45579-1

**Published:** 2023-11-20

**Authors:** Martina Martorell-Barceló, Marco Signaroli, Margarida Barcelo-Serra, Arancha Lana, Eneko Aspillaga, Amalia Grau, Robert Arlinghaus, Josep Alós

**Affiliations:** 1 Instituto Mediterráneo de Estudios Avanzados (IMEDEA, UIB-CSIC), Esporles, Balearic Islands Spain; 2 IRFAP LIMIA (Laboratorio de Investigaciones Marinas y Acuicultura), Andratx, Balearic Islands Spain; 3 Department of Fish Ecology, Fisheries and Aquaculture, Leibniz Institute of Freshwater Ecology and Inland Fisheries, Berlin, Germany; 4 Division of Integrative Fisheries Management, Faculty of Life Sciences, Humboldt Universität zu Berlin, Berlin, Germany

**Keywords:** Behavioural ecology, Ichthyology, Animal behaviour

## Abstract

Chronotypes, the individual differences in daily activity timing, have profound associations with numerous physiological processes. Despite this, the covariance between chronotypes and other aspects of an individual's behaviour has been infrequently explored in non-human animals. This study delves into individual's variation across four axes of personality in a controlled environment, utilising the pearly razorfish, a model species for fish chronotype studies. We identified behavioural types across the aggressiveness continuum and established behavioural syndromes amongst exploration, activity, and boldness, irrespective of body size and condition. Subsequent to this, the experimental subjects were reintroduced to their natural habitat and individually tracked using high-resolution technology to ascertain their chronotypes. Our results revealed that whilst the exploration-activity-boldness syndrome bore no correlation with chronotypes, a significant association was observed between aggressiveness and chronotype. Hence, individuals with later awakening times and rest onsets were more aggressive than their counterparts with earlier awakening times and rest onsets. This study provides pioneering evidence linking fish chronotypes with other behavioural traits, such as aggressiveness, suggesting that behavioural variation could be potentially linked to the individuals' variation in internal clocks and the environmental variables influencing their expression.

## Introduction

Daily activity rhythms in nearly all organisms are synchronised with the 24-h solar cycle through a combination of internal processes (circadian rhythms) and environmental signals or zeitgebers^1^. Differences among individuals of the same species define chronotypes and emerge from individual deviations of the circadian rhythm. These deviations are represented by the parameter tau (τ) resulting in a free-running phase in the absence of zeitgebers and it is modulated by the physical or social environment^2^. In humans, chronotypes are typically defined along the morningness-eveningness continuum. Subjects identified as morning-type, larks, wake up and go to rest early, reaching their mental and physical peak early in the day. Oppositely, evening-type subjects, owls, wake up and go to rest later and perform at their best later in the evening^3^. In general, chronotypes have been widely studied in human and terrestrial animals, while for aquatic animals, like fish, most works have been focused on laboratory studies with captive-reared zebrafish (*Danio rerio*)^4^, limiting our understanding of the ecological implications of fish chronotypes.

While the internal clocks imposed by the circadian rhythm can only be studied under a controlled environment (particularly through shifting light regimes), the study of chronotypes demands information on the daily activity of the individual in their natural habitat^5,6^. Human chronotypes, for example, are usually quantified using a combination of activity telemetry devices to record sleep measurements and questionaries to reveal individual preferences^7^. In terrestrial animals, activity patterns can be measured with different methodologies^8^. However, in aquatic systems, the technical limitations of tracking free-living individuals have restricted our ability to explore how circadian rhythms interact with the environment to form chronotypes. In the past decades, monitoring the behaviour of large numbers of free-ranging fish has become possible due to the development of high-resolution acoustic telemetry^9^. In fact, Alós et al.^10^ used classical acoustic telemetry to evaluate the repeatability of chronotypes of the pearly razorfish (*Xyrichtys novacula*). And more recently, Martorell-Barceló et al.^11^ utilised a high-resolution tracking system to examine how environmental factors modulate the chronotypes of the pearly razorfish. These findings demonstrate that this technology is a valuable tool for studying circadian-related behavioural variation directly in aquatic environments.

Contrary to chronotypes, fish personality has been extensively studied. Animal personality or behavioural types, defined as individual behavioural differences consistently observed over time and across ecological contexts, are described by repeatability scores, a standardised measure to evaluate the consistency of between-individual differences^12–14^. In fishes, behavioural types are usually described along five main axes^15^: (i) Exploration, as the individual's willingness to engage with new situations; (ii) Activity, as the individual's frequency of movement; (iii) Boldness, as the individual's response to a risky situation; (iv) Aggressiveness, as the agonistic response towards conspecifics; and (v) Sociability, as the individual's social interactions, excluding the antagonistic ones^15^.

Far from being anecdotal, fish behavioural types play a part in many ecological processes and have significant consequences for the ecology and evolution of fish populations^16^. Behavioural traits often covary, generating behavioural syndromes^17^. Many examples of behavioural syndromes demonstrate their importance on fitness, ecology, and evolution of fish populations^15^. Behavioural syndromes are related to growth and metabolic rate, maturation, physical condition, and ultimately, life history^18^. In general, proactive, or fast pace-of-life individuals, are more exploratory, active, bold, and aggressive, have higher metabolic rates, develop and mature faster, but have shorter lifespans. In contrast, reactive or slow pace-of-life individuals, are shyer, less exploratory, less active, and less aggressive, have lower metabolic rates, develop and mature more slowly, but live longer^19^. Despite these differences, the reproductive success of both ends of the syndrome (proactive and reactive individuals) is similar, and both strategies can be maintained within the same population^20^.

The variation in circadian-related behaviours or chronotypes can be directly linked to many aspects of an individual's physiology and other behavioural traits, including personality traits. In humans, there are some examples of chronotype-personality behavioural syndromes. For instance, evening-type preference has been related to openness and morning-type preference with conscientiousness and agreeableness^21^. Furthermore, genetic variants of the circadian clock gene period 3 (*PER3*), often related to individual differences in circadian rhythms, have also been linked to extraversion ^22^. Finally, evening-type women, who woke up later, were more aggressive than their morning-type counterparts^23^. In fish, early-active (measured as the peak of activity) zebrafish larvae exhibited less overall activity and were less bold than late-active larvae^24^. This experiment was conducted in the laboratory and did not evaluate naturally expressed chronotypes. However, the evidence found in humans and also in fish reveals that behaviours like the ones used to define personality and chronotypes could covary and be part of the same behavioural syndrome and depend on each other. Despite this, the chronotype-personality syndrome has rarely been evaluated in non-human animals. In fish, the lack of studies on this syndrome is partly due to the general scarcity of chronotype studies in wild fish (to our knowledge, only Alós et al.^10^ and Martorell-Barceló^11^ evaluated this trait in a wild population).

The objective of this study was to investigate whether there is a correlation between behavioural types and chronotypes in a wild marine species or whether they form independent axes of the behavioural structure of individuals. To achieve this objective, we first assessed behavioural types and syndromes in controlled laboratory conditions using classical behavioural metrics such as exploration, activity, boldness, and aggressiveness using automatic and unsupervised analysis by continuous video recording. Secondly, we monitored individuals in their natural environment to measure their chronotypes using state-of-the-art tracking technology. Based on previous work in zebrafish^24^, we can hypothesize the early-type pearly razorfish to be less active, and more aggressive than the later-type individuals. By combining measurements in laboratory and natural settings, we aimed to gain a more comprehensive understanding of behaviour and its implications in marine fish.

## Methods

### Study species and site

The pearly razorfish is a small-bodied wrasse (Labridae) that inhabits the sandy bottoms of temperate waters of the Mediterranean Sea and Atlantic Ocean^25^. This species is a protogynous monandric hermaphrodite, with evident sexual dimorphism showing differences in the shape of the head and colour patterns, with females presenting a characteristic abdominal white spot^26^. Socially, they form harem-like structures where males defend their territory and the females in it^27^. Different behavioural types have been described in wild populations of pearly razorfish, including spatial-behavioural syndromes^28^ and more recently, aggressiveness-related behavioural types under laboratory conditions^29^. Like other razorfish species, pearly razorfish individuals bury themselves in the sand to rest and protect themselves from predators^30^. Since acoustic emissions are not detected when individuals are buried, this behaviour makes this species a perfect candidate to study day/night rhythms using acoustic telemetry^31^. In fact, the decomposition of circadian-related behavioural variation obtained from acoustic telemetry data has revealed a high repeatability in awakening time and rest onset, describing different chronotypes that fluctuate with the environment^10,11^.

For the present study, we collected the experimental individuals (N = 63) during six separate fishing sessions over consecutive weeks in the Marine Protected Area (MPA) of Palma Bay (Mallorca, Western Mediterranean). Due to the limited capacity of our behavioural scoring set up, we could only accommodate and assess 12 individuals at a time. Each week, a new group of up to 12 individuals (experimental batch) was captured and brought to the laboratory for behavioural scoring. Individuals were captured by fishing using standardised hook-and-line gear. Fish were transported in a 50 L container with a constant oxygen flux from the capture site to the Marine Research and Aquaculture Laboratory (LIMIA). Individuals were measured (total length in cm), weighed (fresh weight in g), visually sexed (as this species shows sexual dimorphism), and housed in isolation in behavioural arenas for one week. Individuals remained undisturbed and unfed in their arenas during the first three days for acclimatisation. On the fourth day, the behavioural experiments began, and the tests were carried out for four consecutive days. During the experimental period, individuals were fed daily with live shrimp (2% of their weight) at the end of the last behavioural test. After a week in the laboratory for behavioural scoring, fish were tagged with acoustic transmitters and transported back to the capture location, where they were released to characterise their chronotypes in the wild.

### Description of experimental arenas for behavioural scoring in controlled laboratory conditions

**Figure 1:**
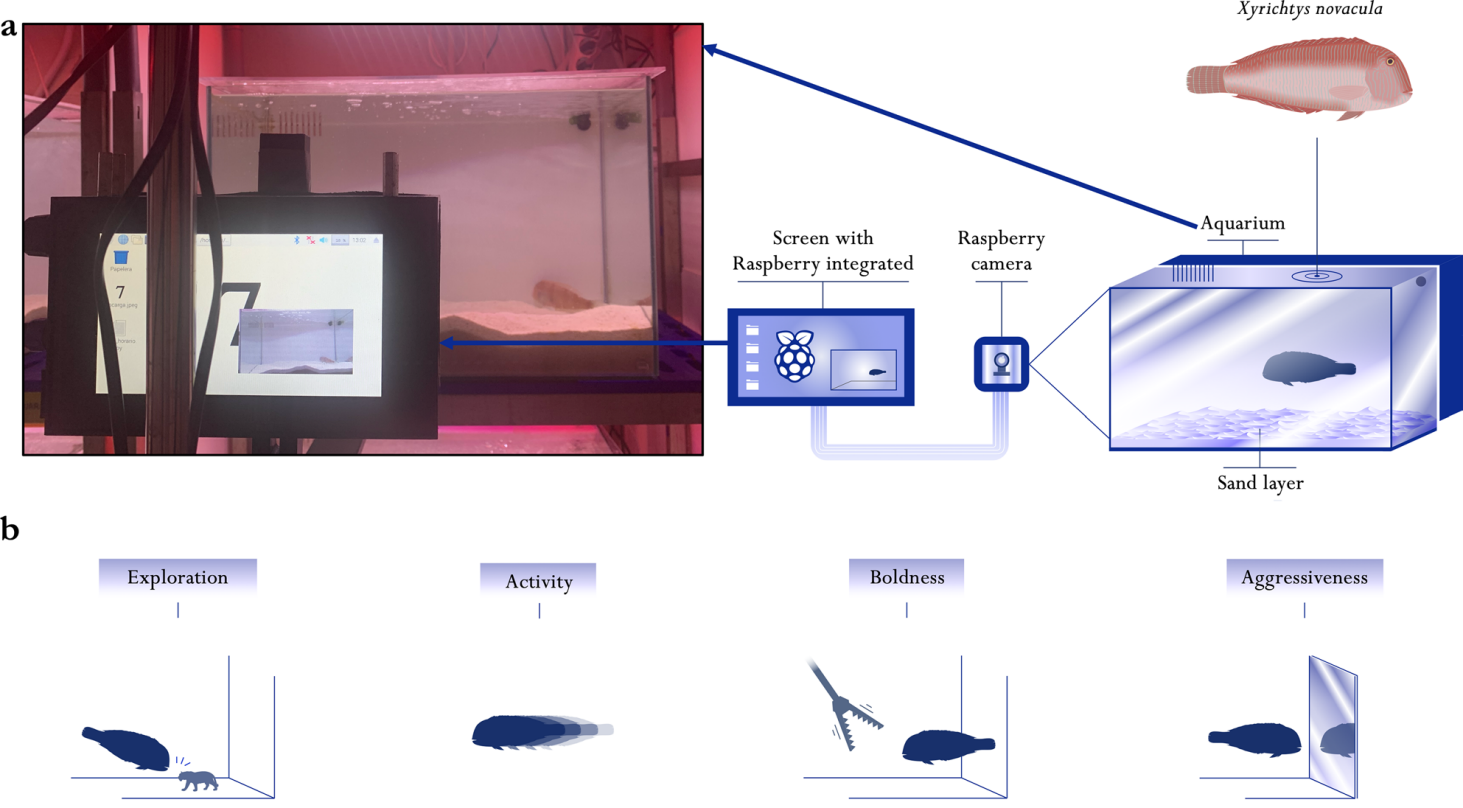
Experimental behavioural assessments in laboratory conditions. Diagram of the experimental behavioural arenas, monitoring equipment (**a**), and tests performed (**b**) to measure four axes of behaviour (exploration, activity, boldness, and aggressiveness) under laboratory conditions in the pearly razorfish (*Xyrichtys novacula*). The recording system (**a**), positioned in front of the aquarium, provided a frontal view of the behavioural tests. This system comprised a Raspberry Pi 3 Model B + computer and a Raspberry Pi Camera Module v2. The four diagrams (**b**) illustrate the behavioural tests conducted (see text). The photo shows the screen of the Raspberry Pi recording a pearly razorfish individual in its aquarium. (Diagram by Javier Sanllehi Hansson).

Fish were tested in experimental arenas (120 L aquaria) with a closed recirculating seawater system (Fig. 1a). Each aquarium contained approximately 5 cm of sand with a specific granulometry (0.5–12 mm) to simulate the habitat of the pearly razorfish and enable the fish to use the sand as a refuge. Including the appropriate substrate providing natural shelter reduced stress and allowed the implementation of specific behavioural testing (boldness test, see below). The closed-water system facilitated temperature control and aquarium maintenance throughout the experimental period. We placed an EHEIM powerLED+^®^ light display on top of each behavioural arena to create optimal light conditions for the pearly razorfish and the recording system (see below), with 12 h of light and 12 h of dark with sunrise set at 7:00 and sunset at 19:00, following Martorell-Barceló et al.^29^. We maintained the water temperature constant at 21 °C (mean and s.d. 21.1 ± 1.8 °C) with a water heater located in the sump of each aquarium.

All individuals' movements were continuously recorded using Raspberry Pi-based systems attached to the front of each aquarium (see details in Signaroli et al.^32^). From the videos obtained, we extracted 18,559 frames and manually labelled the fish's position on the image. This labelled dataset was used to train and validate a YOLOv5 deep learning algorithm for automatic detection and classification of the fish in the video^33^. The trained model provided accurate positioning data (F1 = 0.97; see Signaroli et al.^32^, for more details). Using the trained neural network, we analysed the recordings of the behavioural tests, automatically extracting the position of the fish (2-dimensional) in six frames every minute.

### Behavioural scoring of personality traits

During the four days following the acclimatisation period, we conducted standardised behavioural tests daily to obtain repeated measurements on exploration, activity, boldness, and aggressiveness (Fig. 1b). All individuals were subjected to daily tests with a half-hour break between each test. To minimise potential impacts related to the time of day, we randomised the commencement of these experiments within the morning hours (8:30–12:30). Exploration and aggressiveness tests lasted for one hour each, and the order of these tests alternated daily to prevent intra-day temporal effects. The boldness test, which required active disturbance of the individual by a researcher and the introduction of a food item (see below), was consistently conducted after the exploration and aggressiveness tests. The activity test, lasting two hours, was performed after all other tests, ensuring sufficient recovery time for the individual after the boldness test.

**Figure 2:**
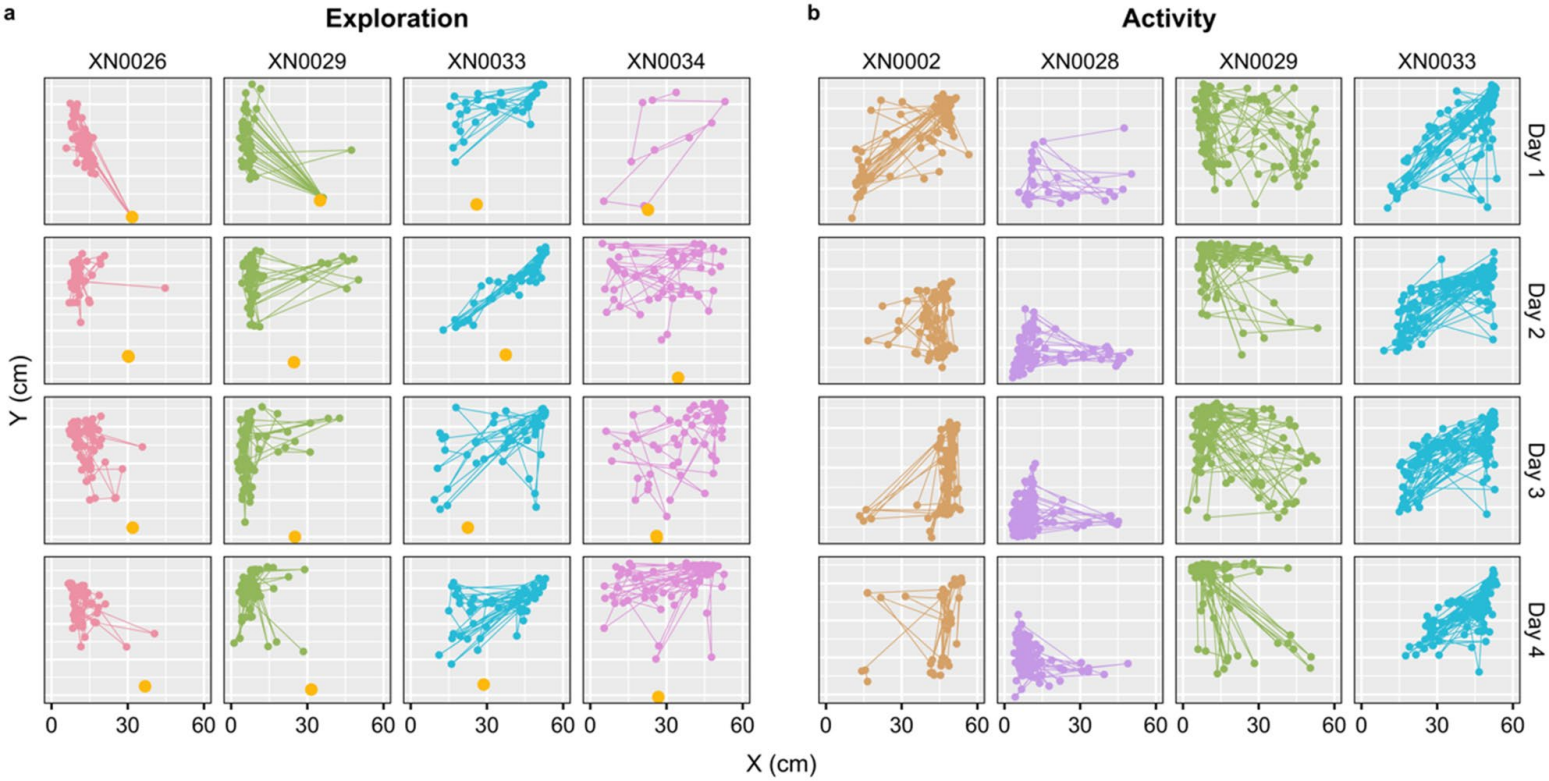
Illustration of an individual's daily minute-by-minute positions (2D), as determined by the deep learning algorithm. Each panel reflects a frontal view of the aquarium, with distances represented in cm. (**a**) The location per minute (colour dots) for each individual (column; ID = XN0026, XN0029, XN0033, and XN0034) during the exploration experiment (1h). The yellow dot signifies the position of the novel object introduced into the aquarium. (**b**) The location per minute (colour dots) for each individual (column; ID = XN0002, XN0028, XN0029, and XN0033) during the activity experiment (2h). In both panels, each row represents the four consecutive experimental days.

Exploration is defined as the individual’s propensity to investigate new objects^19^. We introduced a small toy (a novel object) at the centre of the behavioural arena adjacent to the frontal glass and quantified the fish's response to this new element over the course of an hour. Two types of metrics were used for this purpose: (i) the number of bites (*Interactions*) to the toy, which were manually counted by viewing the video recordings; (ii) three measures extracted through the two-dimensional position of the fish (Fig. 2a), including the time spent outside the sand (refuge) in seconds (*TimeOut*), the minimum distance to the toy in cm (*MinDistance*), and the total time spent near the toy (within a 6 cm radius of the toy's centroid) in seconds (*TimeToy*). These measures are considered reflective of the individual's innate curiosity^34^.

Activity is defined as a measure of the frequency of an individual's movement^19^. We conducted an open-field test, quantifying the total distance travelled and the area covered during the test's two-hour duration. Several metrics were computed from the positional output (XY data) provided by the deep learning algorithm (Fig. 2b). These metrics included: (i) the total distance travelled in m (*Distance*), calculated as the cumulative distances between consecutive positions every minute; (ii) the total area utilised within the behavioural arena in m^2^ (*Area*), defined as the area with a 95% probability of containing the fish; (iii) the core area utilised in m^2^ (*CoreArea*), defined as the area with a 50% probability of containing the fish; (iv) the average turning angle in radians (*MeanAngle*), indicating the preferred turning direction; (v) the variance of the turning angles in radians (*KappaAngle*), calculated under the assumption of a Von Mises circular distribution; and (vi) the time spent outside of the sand (refuge) in seconds (*TimeOut*). *Area* and *CoreArea* were computed using the function kernelUD from the R package adehabitatHR^35^, while *MeanAngle* and *KappaAngle* were calculated using the functions circ.mean and est.kappa from the R package CircStats^36^. Turning angles were computed between consecutive positions, fitting these to the Von Mises probability distribution. All metrics were calculated for individuals detected outside the sand for a minimum of 20s per test, ensuring a representative sample size.

Several methods have been proposed to quantify behaviours related to boldness. However, responses to risky situations are regarded as the most effective method for explaining the ecological implications of boldness^37^. We simulated a predator attack by chasing the fish for 5s with submerged tweezers as a threatening object. Following the simulation, we introduced a food item into the aquarium. The boldness test was always performed last, allowing individuals an unlimited amount of time to emerge from the sand and eat the food item. By analysing the videos, we calculated the emergence latency (*Latency*)—the time it took for individuals to fully emerge from the sand in seconds—as an indicator of boldness. In this case, lower scores denote bolder individuals, while higher scores indicate shyer ones. When a fish did not bury itself in the sand, its boldness score was assigned a value of 0. Given that this is a standard measure for assessing boldness, we did not transform the values.

Lastly, aggressiveness is defined as a measure of an individual's agonistic response towards their own species. We used the mirror test to quantify aggressiveness, as its results are comparable to behavioural responses in natural conditions^38^. We also deemed this test suitable for our species because the individuals responded to the stimulus and did not demonstrate the ability to recognise themselves in front of the mirror^29^. We measured: i) the number of bites (*Bites*), defined as the number of times the individual bit the mirror. A bite was counted when the fish approached the mirror with its mouth open; ii) the number of rams (*Rams*), described as rapid approaches with physical contact to the mirror but with the mouth closed; and iii) the number of charges (*Charges*), described as a fast swim towards the mirror without direct contact. Behavioural scoring was done manually by reviewing the videos.

### Behavioural scoring of the chronotype in free-living fish

We used a novel implementation of a high-throughput acoustic tracking system capable of studying fish chronotypes in hundreds of individuals from the same natural population^39^. At the end of the experimental week in the laboratory, the fish were tagged with Lotek L-AMT transmitters. For the implementation of the tags, individual fish were initially removed from their experimental arenas and submerged in a tank with water containing an anaesthetic solution (0.1 g/L of tricaine methanesulfonate). Upon anaesthetisation, an abdominal incision was made, allowing for the insertion of the tags. Following this, the incision was meticulously sutured. After the procedure, the fish was placed in a recovery tank filled with clean water. Subsequently, they were transported back to their original capture location within the Palma Bay MPA. The tagged fish were monitored using an array of 70 WHS-4250L receivers (Lotek Wireless Inc.) installed in the study area (for more details on the surgical procedure and the acoustic telemetry setup, see Aspillaga et al.^39^). The acoustic data (time series of detections) obtained from the acoustic tracking experiment was imported into the R computing environment^40^. Following Alós et al.^10^, we discretised the acoustic detection time series into 5-min intervals, calculating the number of detections registered within each interval. We then fitted a Hidden Markov Model (HMM) to automatically assign one of two behavioural states, rest (R) or active (A), to each interval. We transformed the observed detection pattern into a Markov chain of detections, assigning a behavioural step to each interval using a Markovian two × two probability transition matrix (A → A, A → R, R → R, R → A)^41^. We used a Zero-inflated Poisson HMM using the R package ziphsmm^42^. From the HMM, we computed the awakening time relative to sunrise and the rest onset relative to sunset. Both metrics have been traditionally used to quantify chronotypes in human and non-human species^43,44^.

Finally, we extracted the individual behavioural score (random effects) to characterise the chronotype of each individual by fitting a Linear Mixed-Effects Model (LMM) for awakening time and another for rest onset. This helped to decompose the raw phenotypic variance into between- and within-individual variances, following the recommendations by Dingemanse & Dochterman^13^. We fitted sex, light, temperature, and waves as fixed effects and individual as a random effect. Light and temperature values were obtained from a Hobo Pendant® (Pendant Temperature/Light 64K Data Logger) placed in the receiver network, while wave data was provided by the oceanographic buoy of the Balearic Islands Coastal Observing and Forecasting System (SOCIB, https://www.socib.es/) located close to the study area (~ 3 km). We then calculated the behavioural scores of each individual as the predicted mean of their behavioural expression. We calculated the standard deviation for each level of the random effect with the repeated samples of the posterior distribution of our model, following the procedure of Hertel et al.^45^ using the function REsim within the R package merTools^46^.

### Data analysis

#### Principal component analysis

We extracted various behavioural metrics for each of the four laboratory-based traits. To investigate patterns of co-variation among these metrics and identify the ones that best explained each trait, we conducted a Principal Component Analysis (PCA) on the normalised and mean-centred variables following the approach described by Martorell-Barceló et al.^29^.

**Figure 3:**
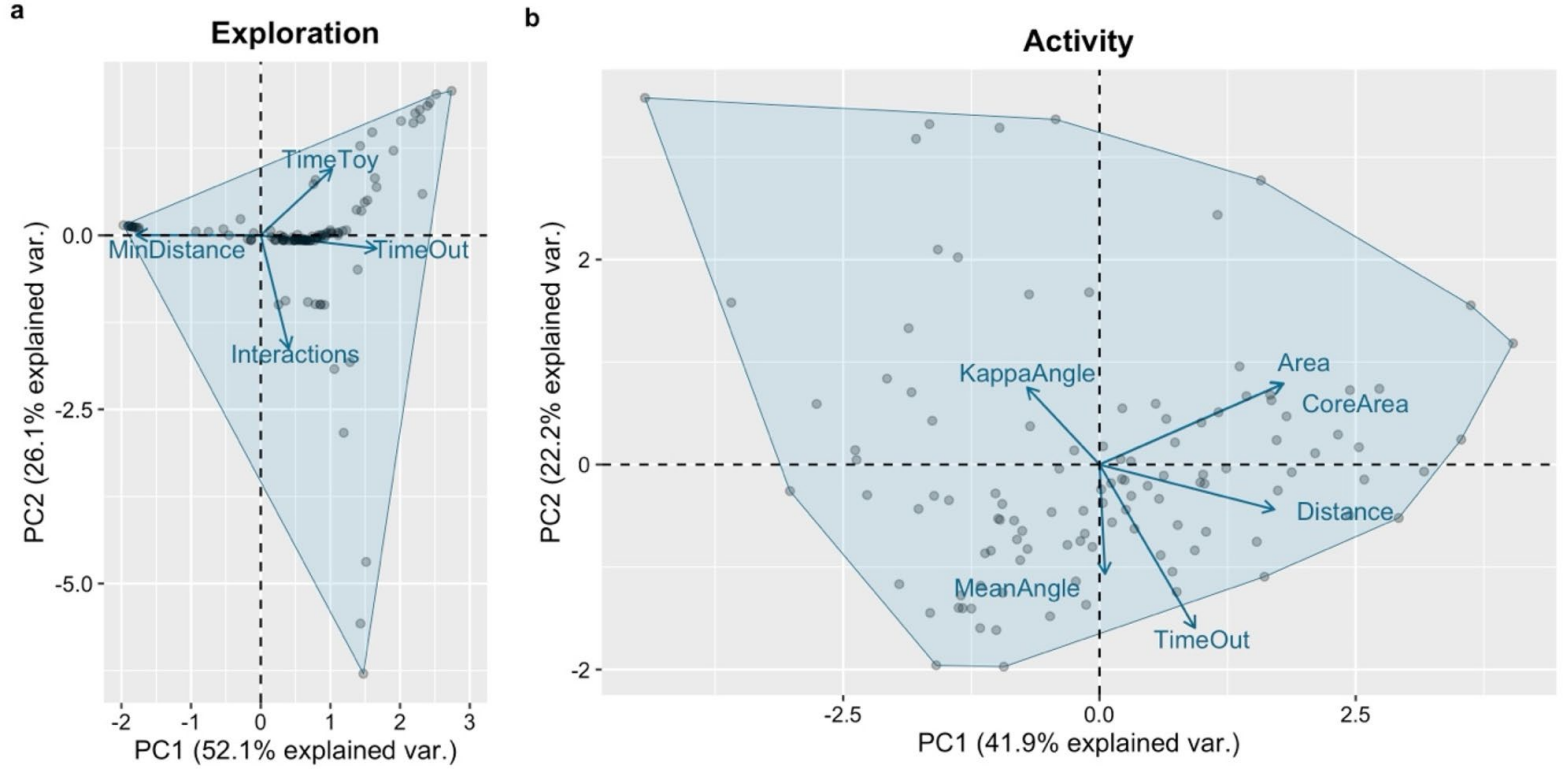
PCA representation. Representation of the two principal components (PC1 and PC2) of the Principal Component Analysis (PCA) for exploration (**a**): *TimeToy* in seconds, *TimeOut* in seconds, *Interactions* and *MinDistance* in centimeters; and for activity (**b**): *Distance* in meters, *Area* in square meters, *CoreArea* in square meters, *MeanAngle* in radians, *KappaAngle* in radians, and *TimeOut* in seconds (refer to the main text for a detailed explanation of each metric and the methods used for their measurement). Each dot represents an observation, and all variables were normalized and centered prior to conducting the PCA.

For exploration, the first principal component (PC1) accounted for 52.1% of the total observed variance (Fig. 3a). Higher scores on PC1 indicated individuals with a greater inclination for exploration, spending more time outside the refuge, more time interacting with the toy, and approaching it from shorter distances. Regarding activity, PC1 explained 41.9% of the total observed variance (Fig. 3b). Higher PC1 values represented more active individuals, characterized by greater movement within larger areas, covering longer distances, and spending more time outside the refuge. Lastly, for aggressiveness, PC1 explained 87.7% of the observed variance and was primarily linked to the *Bites*. Throughout the literature, higher scores in the mirror test denotate more aggressive individuals. To aligning our PC1 values with this description, we adjusted the PC1 values to natural numbers by multiplying them by −1, so that higher values reflected individuals that exhibited more frequent biting behaviour towards the mirror (for further details, see Martorell-Barceló et al.^29^). These PC1s were employed as scores for their respective traits and subsequently utilised as response variable in the following statistical models (see below).

#### Adjusted repeatability and contextual variance for the laboratory-based behaviours

To analyse the behavioural data, we used two datasets: (i) the laboratory dataset, which included individuals with at least two days of data from laboratory trials, and (ii) the laboratory and wild dataset, which included individuals with at least two days of laboratory data and 7 days of tracking data in the wild. It is important to note that our aim was to include four trials for each test in the laboratory dataset; however, due to various technical issues, it was not possible to obtain four trials for all individuals. For subsequent analyses, we only include individuals with a minimum of two trials for each laboratory-based trait.

We employed Linear Mixed-effects Models (LMMs) to partition the variance of the four laboratory-based behaviours into individual, contextual, and population components in order to calculate repeatability scores and define behavioural types. Repeatability (*R*) score provides an estimate of individual variation within the population and allows us to assess consistent between-individual differences^14^. *R* score was calculated as the ratio of between-individual variance (*V*_*ind0*_) to the sum of  *V*_*ind0*_ and within-individual variance (*V*_*e0*_)^13,14^. To account for the influence of contextual variables on specific behavioural traits, we computed adjusted-*R* scores. We used the R library lme4^47^ to fit four separate LMMs, one for each trait, with fixed factors including body size, condition, and day of trial, and random factors including individual and experimental batch (see details below). Condition, which represents the internal state of the individual, was calculated as the ratio of the predicted weight for the species to the fresh weight (in g; see Martorell-Barceló et al.^29^ for more information). Experimental batch (determined by the limited number of experimental arenas) was included as a random factor.

The fixed structure of the LMMs was scaled and normalized. We assessed the normality of residuals and, if necessary (for boldness and aggressiveness), logarithmic transformations were applied to meet the assumption of normality. Model reduction was performed using bidirectional elimination, a combination of backward and forward stepwise selection, until the lowest Akaike Information Criterion (AIC) was reached (with a cut-off of two points in the process, see Supplementary Table S1). Confidence intervals (CI) for all parameters were estimated at 2.50% and 97.50%. The significance of adjusted-*R* scores was determined using the constricted model (without random effects), considering a reduction of more than 2 in the AIC between the constrained and unconstrained models as significant^10,48^.

#### Correlations between laboratory-based and wild-based behaviours and emergence of behavioural syndromes

To assess the influence of chronotypes on personality, we reanalysed our four LMMs using the laboratory and wild dataset. Fixed factors included body size, condition, day of trial, awakening time, and rest onset, while individual and experimental batch were treated as random factors. Subsequently, we employed Multivariate Generalized Linear Mixed Models (MGLMMs) to examine the phenotypic correlations (r_phe_) between pairs of laboratory-based behavioural traits and decompose them into between-individual (r_ind_) and within-individual (r_e_) correlations. Analytical details confirmed that only r_ind_ accurately captures behavioural syndromes^49^. We screened for pairwise behavioural syndromes, such as exploration-activity, exploration-boldness, exploration-aggressiveness, activity-boldness, activity-aggressiveness, and boldness-aggressiveness, utilizing the MCMCglmm library in R following the protocol established by Dingemanse and Dochtermann^13^. Significant variables identified in the models were included in the analysis of behavioural characterization. In cases where variables did not exhibit a statistically significant effect on behaviour, awakening time and rest onset were incorporated to investigate the impact of chronotypes on behavioural syndromes. Confidence intervals (CI) were estimated to determine the significance of correlations, and when the estimate did not encompass zero, the correlated traits were considered to form a behavioural syndrome.

### Ethical notes

This study received a positive evaluation from the Ethical Committee for Animal Experimentation of the University of the Balearic Islands (references for the protocols CEEA 324 107/01/19 and CEEA 97/0718). It was approved by the Department of Fisheries of the Government of the Balearic Islands, which issued permits for the capture of wild animals and the installation of the acoustic receptor network within the MPA (ref. 2019/20AEXP). All procedures were conducted by trained and competent personnel in accordance with the European Directive (2010/63/UE) transposed into Spanish law (RD53/2013), ensuring the implementation of good animal care, health, and welfare practices. Tagged animals were followed using acoustic telemetry and displayed normal behaviour within few days of release, providing with evidence of survival and minor effects of the surgical procedure. Standardized personality tests were performed at LIMIA, an authorized facility for animal housing and experimentation (REGA ES070050000839), no animals were lost during the experimental trials and were all released back to the capture location after behavioural testing. Our study has been reported in accordance with the ARRIVE guidelines^50^.

## Results

### Sample size and characteristics

We considered the data for posterior analysis when the experimental trial for a particular behavioural axis was conducted on the same individual on at least two different days. In summary, a mean of 42.5 ± 5.07 individuals, with a mean size of 15.30 ± 2.43 cm, weight of 42.63 ± 20.48 g, and condition of 1.37 ± 3.16 were successfully scored in the laboratory. Additionally, a mean of 25.75 ± 3.3 individuals with a mean size of 14.67 ± 2.21 cm, weight of 37.81 ± 18.06 g, and condition of 1.11 ± 2.74 were successfully scored both in the laboratory and in the wild (Table 1).

**Table 1:** Summary of the behavioural datasets.

Dataset		N	Size	Weight	Condition	Observations
Exploration	Laboratory	44	15.37 ± 2.51	43.52 ± 21.25	1.68 ± 3.25	3.22 ± 0.71
	Lab + Wild	28	14.70 ± 2.21	38.08 ± 17.69	1.11 ± 2.71	3.43 ± 0.69
Activity	Laboratory	39	15.55 ± 2.54	45.44 ± 21.7	1.32 ± 3.95	3.18 ± 0.73
	Lab + Wild	22	14.81 ± 2.40	39.54 ± 19.87	0.94 ± 2.99	3 ± 0.74
Boldness	Laboratory	38	15.65 ± 2.56	46.31 ± 22.12	1.4 ± 3.56	2.95 ± 0.82
	Lab + Wild	24	14.83 ± 2.26	39.36 ± 18.38	0.90 ± 2.82	3.42 ± 0.72
Aggressiveness	Laboratory	49	15.25 ± 2.45	42.81 ± 20.53	1.27 ± 3.67	3.22 ± 0.75
	Lab + Wild	29	14.71 ± 2.15	38.05 ± 17.39	1.08 ± 2.68	3.78 ± 0.81

The first column represents the datasets for different behavioural trials: Exploration, Activity, Boldness, and Aggressiveness. The datasets are divided into the laboratory-only dataset and the dataset including both laboratory and tracking data in the wild (Lab + Wild). N denotes the number of individuals included in each dataset, with a minimum requirement of being tested at least twice in the laboratory and having 7 days of data at sea. Size and Weight indicate the mean and standard deviation (s.d.) of the total length (in cm) and weight (in g), respectively. Condition represents the mean and standard deviation (s.d.) of the condition, which serves as a measure of the internal state (refer to the main text for detailed information on how it was calculated). Observations denote the average number of behavioural observations per individual (days), ranging from 2 to 4.

### Behavioural types and contextual variance

**Figure 4:**
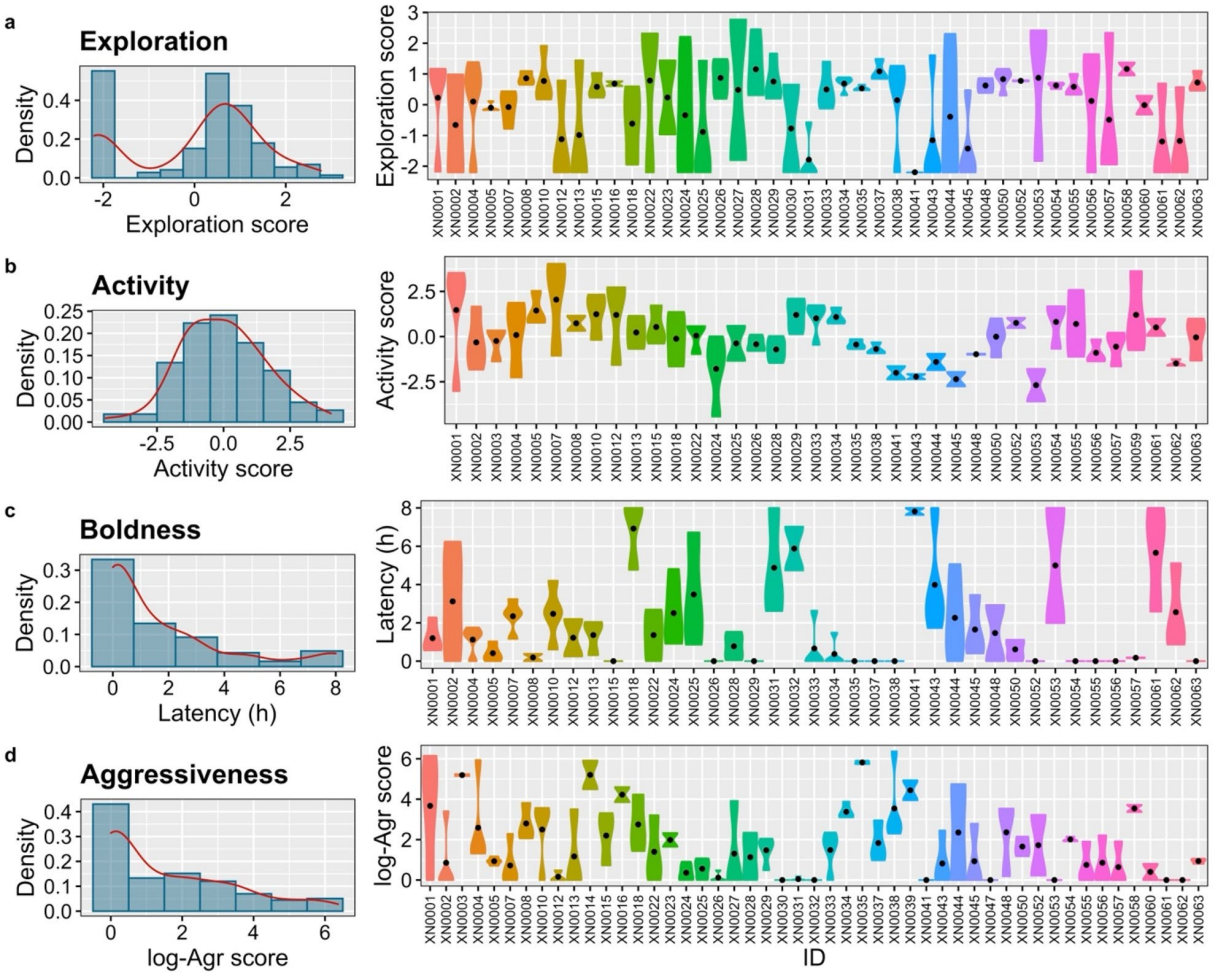
Behavioural types. Density population plots (left column) and daily individual variation plots (violin plots in the right column) for (**a**) exploration, (**b**) activity, (**c**) boldness, and (**d**) aggressiveness (abbreviated as Agr) in laboratory-based experiments on pearly razorfish, *Xyrichtys novacula*. Each colour in the violin plots represents an individual, with the size of the figure indicating the degree of individual variation, and a black dot denoting the mean.

We obtained an adjusted-*R* score of 0.16 [0.11–0.21] for exploration, which was deemed significant based on the comparison of constrained and unconstrained models, fulfilling the criterion for behavioural types (Table 2). The best model, as indicated by the lower AIC, only included the day of trial, suggesting that exploration-related behavioural types emerged independently of body size (and sex), and condition. The day of the trial showed a significant and positive effect, indicating an increase in exploratory behaviours over the course of experimental days (Table 2). Our findings demonstrate substantial variation in exploration among pearly razorfish (Fig. 4a). The most exploratory individual (ID = XN0028) spent an average of 17.55 ± 25.61 min near the toy, 52.72 ± 6.07 min outside the sand, approached the toy to a minimum distance of 4.81 ± 5.01 cm, and bit the toy 1.15 ± 0.5 times. In contrast, the least exploratory individual (ID = XN0041) did not spend any time near the toy, only spent an average of 14.48 ± 28.57 min outside the sand and had a minimum distance to the toy of 29.94 ± 29.94 cm (Fig. 4a).

**Table 2:** Behavioural types.

	Estimate	l-CI	u-CI	Significance
Exploration				
Intercept	0.01	−0.26	0.29	0.920
Day	0.34	0.12	0.55	0.002**
Adjusted-*R*	0.16	0.11	0.21	
AIC = 516.95 (AICc = 514.35)				
Activity				
Intercept	−0.04	−0.41	0.33	0.840
Adjusted-*R*	0.24	0.17	0.33	
AIC = 422.69 (AICc = 424.91)				
Boldness				
Intercept	6.28	5.35	7.21	< 0.001***
Adjusted-*R*	0.67	0.60	0.73	
AIC = 541.47 (AICc = 467.48)				
Aggressiveness				
Intercept	1.63	1.29	1.97	< 0.001***
Adjusted-*R*	0.53	0.45	0.61	
AIC = 599.38 (AICc = 639.39)				

Parameters and confidence intervals (CI) [lower (l-) and upper (u-)] for the four LMMs fitted to laboratory-based behaviours. Significance is indicated by the p-value and the following codes: 0.001 '***' 0.01 '**' 0.05 '*'. The LMMs presented in this table have been reduced based on the Akaike Information Criterion (AIC). Additionally, the table provides the estimates of adjusted-*R* for each trait, AIC reduced (AIC), and the AIC for the constrained model (AICc).

For activity, we obtained an adjusted-*R* score of 0.24 [0.17–0.33], which was considered significant, indicating the presence of behavioural types in activity as well. The best model, with the lower AIC, excluded fixed effects and only included the individual as a random effect, suggesting that activity-related behavioural types emerged independently of body size (and sex), condition, and day of trial (Table 2). The most active individual (ID = XN0007) travelled an average distance of 842.13 ± 334.93 m, covered an area of 171.15 ± 107.15 m^2^, had a core area of 48.2 ± 32.49 m^2^, and spent 114 ± 2.70 min outside the sand. In contrast, the least active individual (ID = XN0045) travelled an average distance of 34.75 ± 43.55 m, covered an area of 45.89 ± 64.66 m^2^, had a core area of 11.25 ± 15.87 m^2^, and spent 19.8 ± 0.21 min outside the sand (Fig. 4b).

Regarding boldness, we obtained an adjusted-*R* score of 0.67 [0.60–0.73], which was considered significant, indicating the presence of behavioural types in boldness. The best model, with the lower AIC, excluded fixed effects and only included the individual as a random effect, suggesting that boldness-related behavioural types emerged independently of body size (and sex), condition, and day of trial (Table 2). The most notable differences in boldness were observed between the 11 bolder individuals who were unaffected by the simulated attack and the shyest individual (ID = XN0041), who took an average of 7.80 ± 0.26 h to emerge from the sand after the simulated attack (Fig. 4c).

The adjusted-*R* score for aggressiveness was estimated at 0.53 [0.45–0.61], and similar to boldness and activity, the most parsimonious LMM only included the individual as a random effect (Table 2). These results suggest that aggressiveness in pearly razorfish is independent of body size (and sex), condition, and day of trial (Table 2). The most aggressive individual (ID = XN0035) bit the mirror an average of 332 ± 29.28 times, while the 8 least aggressive individuals did not exhibit any biting behaviour towards the mirror on any day (Fig. 4d).

### Behavioural syndromes

**Figure 5:**
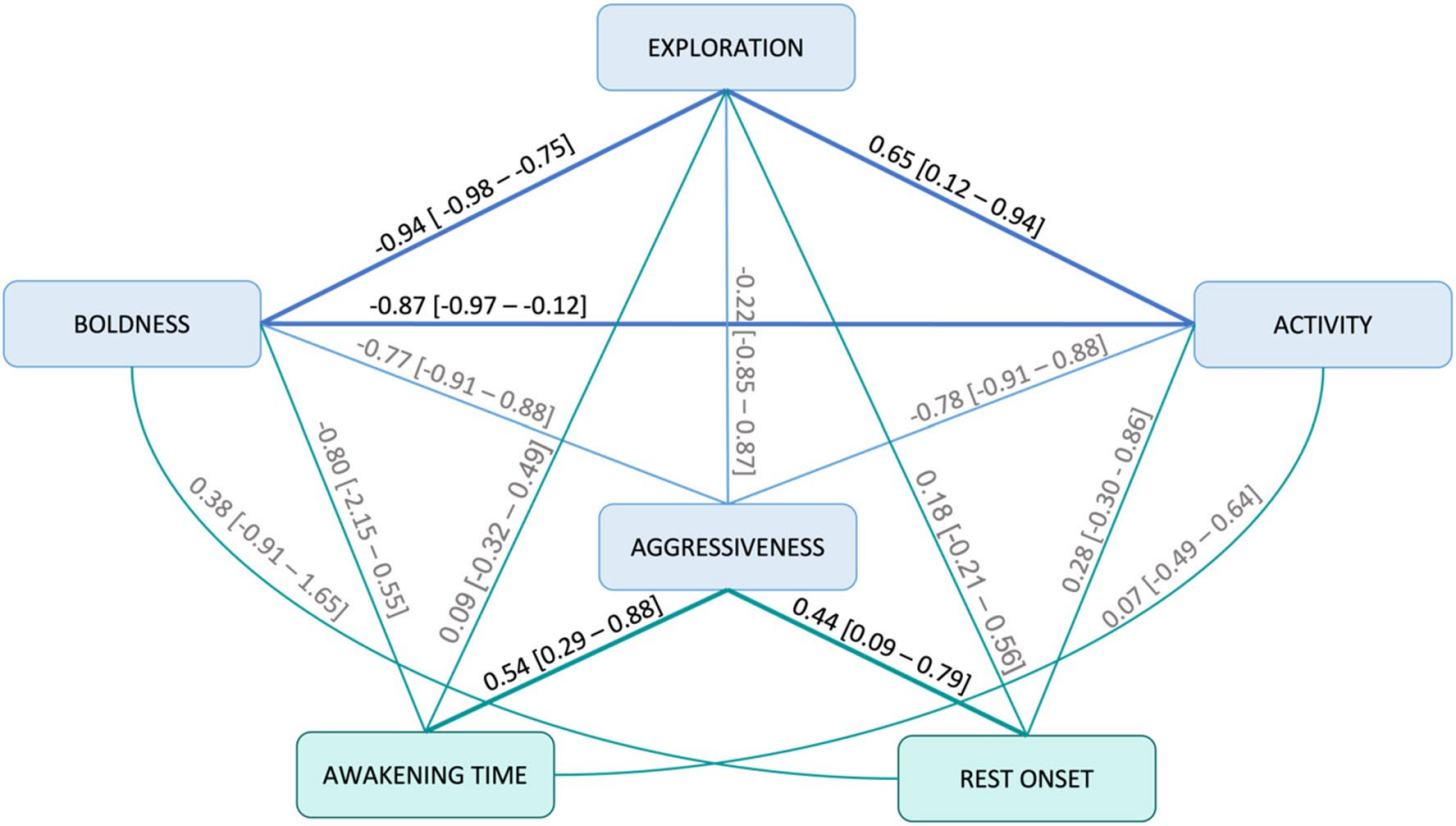
Behavioural syndromes. Diagram illustrating the between-individual correlations (r_ind_) among traits. For chronotypes-personality correlations, the estimates were obtained from the LMMs for each laboratory-based behavioural trait (exploration, activity, boldness, aggressiveness), considering only the effects of chronotypes (activity onset and rest onset). For personality correlations, the estimates were obtained from the MGLMMs. In both cases, the value represents the r_ind_ with their corresponding confidence intervals shown in square brackets. Significant correlations are depicted by thicker lines and values in black, while non-significant correlations are shown with thinner lines and values in grey.

When examining correlations among traits to explore behavioural syndromes, we discovered a significant positive correlation between exploration and activity (r_ind_ = 0.65 [0.22–0.94]), as well as negative correlations between exploration and boldness (r_ind_ = − 0.94 [−0.98 to −0.75]) and between activity and boldness (r_ind_ = −0.87 [−0.97 to −0.12]). Negative correlations with boldness are observed because boldness is measured as latency, and thus lower values indicate individuals that emerge rapidly from the sand. Consequently, we identified a syndrome where individuals with higher exploration tend to be more active and bolder. No significant correlations were observed between aggressiveness and other laboratory-based behavioural traits (Fig. 5).

Regarding the interaction between personality and chronotypes, we observed a positive relationship only between aggressive-related behavioural types and chronotypes. Individuals exhibiting higher aggression levels in the laboratory mirror test demonstrated delayed awakening times and rest onsets compared to less aggressive individuals. However, we did not find any significant correlations between other personality traits (exploration, activity, boldness) and chronotypes (Fig. 5).

## Discussion

In this study, we investigated the personality traits and behavioural syndromes of the pearly razorfish and made significant progress in understanding their behaviour in both wild and laboratory conditions. By comparing measurements from laboratory and field observations, we examined the interaction between different behavioural traits and chronotypes. For the first time in the pearly razorfish, we identified between-individual differences and defined behavioural types for personality traits under laboratory conditions. Our results demonstrate that all the personality traits studied exhibit significant *R* scores, indicating consistent behavioural patterns over time. While previous research had established the *R* of aggressiveness in this species^29^, our study provides the first evidence for behavioural consistency in exploration, activity, and boldness under controlled conditions. Furthermore, our findings suggest an association between aggressiveness and the chronotypes of the pearly razorfish.

The *R* score for exploration obtained in this study (*R* = 0.16) was lower compared to findings in other species (*R* = 0.47^34^). We attribute this lower *R* score to the possibility that individuals became habituated to the presence of novel objects in their arena, despite presenting a new object each day. As our results indicate, exploration was positively influenced by the day of the trial. Frequent exposure to new objects, even if different each time, can lead to familiarity and decreased fear, thereby increasing exploratory behaviour^51^. Nevertheless, our research supports the idea that pearly razorfish populations consist of individuals with varying degrees of exploration, which can have ecological implications. The exploration axis is particularly relevant for species dispersal, as more exploratory individuals are more likely to leave their current location and establish new populations^15^. In the context of pearly razorfish, individuals that are more inclined to leave their territory in search of better territories may exhibit higher levels of exploratory behaviour. However, this carries the risk of losing their current territory or finding that the new territory is not superior in quality to the original one. Conversely, less exploratory individuals face a greater risk of losing their territory but may not gain significant benefits from seeking a better one. The *R* score obtained, suggests that our population exhibits a balanced distribution across the exploration continuum, with each strategy providing different fitness benefits. However, this balance may be disrupted in populations exposed to fishing pressure, since the most exploratory individuals are the most susceptible to fishing^52^. Consequently, fishing pressure can disrupt the balance of the exploration continuum, selecting against more exploratory individuals. This, in turn, may lead to populations comprised of increasingly less exploratory individuals, further isolating them from one another as fishing primarily targets individuals that could potentially connect different areas.

The obtained *R* score for activity (*R* = 0.24) is considerably lower compared to a previous study in the same species (*R* = 0.83^10^), where activity was measured as travelled distance using acoustic tracking in the natural environment. Several factors may contribute to this lower *R* score here. Firstly, the finite space of the aquarium can limit the range of activity for individuals, potentially reducing population variance as some individuals may be constrained by the confined space of the behavioural arenas. Secondly, sample size can influence *R* score estimates^53^, the smaller sample size in the previous study could have inflated this value^10^. Notably, activity is often strongly related to the condition of individuals, with more active individuals typically associated with better foraging and growth rates compared to less active individuals^54^. However, here, the foraging area was small and identical for all individuals, enabling them to utilise resources without requiring extensive movement. Consequently, activity may not have been influenced by the covariates studied, suggesting that it is an important intrinsic factor of an individual's behaviour. Despite the lower *R* score in activity observed here compared to previous works, we have still found evidence that wild populations of pearly razorfish consist of individuals exhibiting different activity levels. This variation in activity can have significant fitness consequences for individuals, as well as ecological and evolutionary implications. For instance, higher activity levels in pearly razorfish can increase the likelihood of encountering a fisher, thus selecting against more active individuals^52^.

The *R* score for boldness was the highest obtained in our study (*R* = 0.67), clearly distinguishing between bold and shy individuals regardless of body size or sex. The *R* score for boldness has been estimated in various fish species, yielding a large range of values, including within species. For instance, three-spined sticklebacks (*Gasterosteus aculeatus*) exhibited *R* scores ranging from 0.26 (mean reported by Bell et al.^55^) to 0.38^56^. These variations in *R* scores, even within the same species, suggest that boldness is heavily influenced by species-specific and population-specific factors. Here, boldness consistently emerged across individuals, independent of size, sex, day of trial, or condition, indicating its intrinsic nature. Boldness has significant implications for an individual's fitness, as it represents a trade-off between acquiring food resources to meet energetic demands and the risk of predator exposure^57^. Bolder individuals may gain increased access to food resources but also face a higher risk of predation, while shyer individuals may have limited access to food but enhance their survival by reducing their exposure to predators^18^. Therefore, our results suggest that individual pearly razorfish consistently adopt either a bold or shy strategy based on their energetic requirements.

The *R* score for the aggressiveness axis in our study (R = 0.53) was relatively high compared to the average aggressiveness *R* scores across traits and taxa (0.37^53^). Numerous studies have estimated different *R* scores for aggressive behaviours in various species. For instance, McGhee and Travis^58^ reported an *R* score of 0.72 for blue catfish (*Lucania godei)*. In contrast, Sbragaglia et al.^59^ did not find a significant *R* score for aggressive behaviours of zebrafish towards a mirror. The discrepancies in *R* scores could be attributed to differences in methodology. The first study used the opponent test to establish dominance rank^58^, while Sbragaglia et al.^59^ used the mirror test and quantified the number of charges. We evaluated the number of charges, but calculated *R* score form an aggressiveness score, derived from a PCA of our measurements, with bites being the most influential metric. Way et al.^60^ calculated the *R* score of bites towards a mirror in zebrafish and reported a very similar *R* score (R = 0.56) to the one we obtained. These examples highlight the sensitivity of the *R* score to the selected aggressiveness metric and test employed. Our findings demonstrate that the mirror test is a reliable and effective method for measuring aggressiveness in pearly razorfish during short assessments. Mirror test enables precise quantification of aggression by measuring real-time agonistic responses to a reflection that emulates the interactions with conspecifics^60^. Our results suggest that pearly razorfish exhibit a consistent level of aggressive behaviour, indicating a potential role for this trait in social interactions by defining social hierarchies or territorial defence^29^.

Behavioural syndromes are a characteristic of a population as they describe the correlation between rank-order differences among individuals over time and across different situations^61^. We identified a behavioural syndrome comprising exploration, activity, and boldness. Furthermore, we discovered that aggressiveness acts as an independent axis of behaviour in the pearly razorfish. While the exploration-activity-boldness syndrome is commonly observed in fish^17^, most studies assessing this syndrome did not specifically evaluate aggressive behaviours, so they were unable to determine if aggressiveness is part of the syndrome. Some studies have explored the relationship between aggressiveness and other specific traits. Only a weak relationship between aggressiveness and activity was shown based on a review of the literature^62^. Aggressiveness is often associated with boldness^63^, but Way et al.^60^ did not detect a link between boldness and aggressiveness in zebrafish and suggested that the aggressiveness-boldness syndrome is more commonly observed in populations facing environmental constraints, such as high predation pressure. For instance, stickleback populations experiencing high predation pressure often exhibit a clear boldness-aggressiveness syndrome, whereas predator-free populations do not display this syndrome. Therefore, the emergence of behavioural syndromes may depend on the ecological context of a particular population^17^. Based on the current evidence, it could be suggested that our study population is not subjected to intense predatory pressure, potentially explaining the absence of the boldness-aggressiveness syndrome in our findings. However, without definitive data on the predatory pressure in our population, further research would be essential to elucidate the ecological reasons of these outcomes. Therefore, our findings suggest that the exploration-activity-boldness syndrome has been favoured in our pearly razorfish population. However, further studies conducted in natural environments are needed to understand the ecological causes and consequences of the emergence of this syndrome in the pearly razorfish.

Traditionally, behavioural syndromes have been defined as correlations between two or more behavioural types^17^. However, this definition can be expanded to include the correlation between the classical axes of personality, such as exploration, activity, boldness, and aggressiveness, with other repeatable behaviours, such as chronotypes^23^. While numerous studies have examined the personality-chronotype syndrome in humans, there is a lack of consistency and even contradictory findings. In fish, early-type larvae of zebrafish exhibited lower overall activity compared to later-types and may display shyer behaviours^24^.

Here, we did not find a relationship between the exploration-activity-boldness syndrome and chronotypes in pearly razorfish. Our findings are not directly comparable to Amin et al.^24^ due to differences in behaviour between the two species or by differences in the methodology. While their study focused on correlations between total daily activity and circadian rhythms measured in the laboratory, we assessed chronotypes in the natural environment. Additionally, a previous study on pearly razorfish found no correlation between awakening times and daily distance travelled, both measured in wild conditions^10^. However, we did observe a relationship between the continuum of aggressiveness and chronotypes. Similar to the findings in humans^23^, later-type individuals of pearly razorfish exhibited higher levels of aggressiveness compared to early-type individuals. This work represents the first evidence of the chronotype-personality syndrome in marine fish. It is important to note that the relationship between chronotypes and personality traits can vary across species and populations, and further research is needed to fully understand the ecological and evolutionary implications of the chronotype-personality syndrome in different organisms. Furthermore, it is important to note the role of the circadian rhythms in stress physiology of fish^64^. These rhythms can influence the secretion of circulation corticosteroids, which may affect the behavioural response of fish. This highlights the need for future experimental studies on fish personality traits to account for daily hormonal fluctuations. Therefore, the relationship between chronotypes and personality may be modulated by physiological and hormonal factors, adding another layer of complexity to understanding the ecological and evolutionary implications of the chronotype-personality syndrome across different organisms.

Chronotypes of pearly razorfish are described along the activity-duration continuum^11^, and there are noticeable differences among individuals, particularly in terms of awakening time. Chronotypes arise from the interaction between the internal clock and the environment, with the timing of activity start and end being genetically constrained^6,43^. In the specific case of pearly razorfish, an individual with an early awakening time can engage in foraging and territory establishment while its neighbours are still buried, avoiding the need for engaging in agonistic interactions. On the other hand, an individual that starts its activity later may need to be more aggressive to secure a territory and access resources. However, aggressive behaviours involve significant energy expenditure^65^. Therefore, an individual with a later activity onset and higher aggression levels may need to extend its activity beyond sunset and endure the associated predation risk to meet its energy demands. Our findings demonstrate a strong association between chronotypes and aggressiveness in pearly razorfish. However, further studies conducted in natural habitats and diverse populations are necessary to obtain a comprehensive understanding of how both traits influence territory formation. It is also important to investigate potential variations within a day and evaluate how these mechanisms support the proposed chronotype-aggressiveness syndrome. Genetic studies could provide insights into the underlying mechanisms of this syndrome. For example, variations in the clock gene period 3 (*PER3*), present also in marine fish^66^, have been linked to behaviours such as extraversion in humans^22^.

Additionally, the role of individual behaviours in determining vulnerability to capture continues to be an intriguing topic, as it involves the interplay of multiple factors. Aggression levels can make individuals more susceptible to capture, suggesting that more aggressive fish may become easier targets^67^. However, the vulnerability to capture in pearly razorfish is significantly influenced by awakening time^68^. Fish that start their activities earlier, due to prolonged exposure to potential threats and higher encounter rates with anglers, exhibit heightened vulnerability. The apparent contradiction between these findings can be attributed to their different focuses. While Sutter et al.^67^ emphasized behavioural traits such as aggression, Martorell-Barceló et al.^68^ concentrated on temporal aspects of behaviour. When viewed together, they provide a more nuanced understanding of what makes an individual more vulnerable to capture: their aggression levels or their chronotype? Our findings, derived from an MPA with fishing restrictions, may be particularly influenced by this specific context. However, they underscore the importance of extending this line of inquiry to natural environments where fishing is permitted. Future research should aim to unravel the intricate relationship between the chronotype-aggressiveness syndrome and vulnerability to capture in pearly razorfish. It would be particularly interesting to compare the relative importance of these two traits in predicting capture vulnerability and explore their potential co-evolution under fishing pressure. Such investigations could provide valuable insights to shape effective fishery management strategies that consider these nuanced behavioural aspects.

## Conclusions

Our study contributes novel insights into the behavioural framework of the pearly razorfish, highlighting the importance of considering multiple personality traits and their interactions when investigating wild marine species. Through our research, we have demonstrated the presence of distinct behavioural types for exploration, activity, boldness, and aggressiveness in this species. We have also revealed significant variation in each of these behavioural traits among individuals. Notably, we have identified a behavioural syndrome wherein individuals exhibiting higher levels of exploration also display increased activity and boldness. Our study also represents the first evidence of a correlation between circadian-related traits and personality in fishes. By combining laboratory observations and tracking individuals in their natural environment, we have elucidated the association between chronotypes and personality traits in the pearly razorfish. This finding contributes to the growing body of knowledge on the interplay between circadian rhythms and behaviour in aquatic organisms. The observed behavioural diversity within pearly razorfish populations underscores the importance of considering individual variation when studying the ecology of this species. Our study provides a foundation for future research to delve deeper into the behavioural patterns and underlying mechanisms that shape the behaviour of the pearly razorfish. Such investigations will further our understanding of the ecological implications of behavioural diversity and contribute to the broader field of animal behaviour research.

### Supplementary Information

Supplementary Table S1.

## Data Availability

https://doi.org/10.20350/digitalCSIC/15301.
