# Supplementary material for: Chronotypes-personality behavioural syndromes in wild marine fish
Source: Sci Rep. 2023 Nov 20;13:20281. doi: 10.1038/s41598-023-45579-1 (PMC10662165; doi:10.1038/s41598-023-45579-1)
Supplement: Supplementary file 1 — Supplementary Table S1. [file 41598_2023_45579_MOESM1_ESM.docx]

Supplementary material of Chronotypes-Personality Behavioural Syndromes in Wild Marine Fish

*Martorell-Barceló et al.*

Table S1. **Original summaries of the Linear Mixed-Effect Models.** Estimates and confidence intervals (CI) [lower (l-) and upper (u-)] for the four LMM fitted to laboratory-based behaviours. Significance is indicated by the p-value and the following codes: 0.001 '***' 0.01 '**' 0.05 '*'. Additionally, the table provides the original AIC (with all variables included as fixed effects; AIC, and the AIC of the reduced model (with only the significant variables, presented in the main text; AIC_R_).

|  | Estimate | l-CI | u-CI | Significance |
| --- | --- | --- | --- | --- |
| EXPLORATION | | | | |
| Intercept | 0.01 | -0.26 | 0.29 | 0.920 |
| Day | 0.34 | 0.12 | 0.55 | 0.002 ^**^ |
| Size | 0.13 | -0.15 | 0.41 | 0.37 |
| Condition | -0.14 | -0.42 | 0.14 | 0.35 |
| AIC = 525.70 (AIC_R_ = 516.95) | | | | |
| ACTIVITY | | | | |
| Intercept | -0.04 | -0.41 | 0.33 | 0.840 |
| Day | 0.21 | -0.06 | 0.47 | 0.12 |
| Size | 0.20 | -0.18 | 0.57 | 0.32 |
| Condition | 0.10 | -0.28 | 0.48 | 0.63 |
| AIC = 431.91 (AIC_R_ = 422.69) | | | | |
| BOLDNESS | | | | |
| Intercept | 6.28 | 5.35 | 7.21 | <0.001 ^***^ |
| Day | -0.10 | -0.45 | 0.26 | 0.60 |
| Size | -0.54 | -1.52 | 0.43 | 0.29 |
| Condition | 0.01 | -0.93 | 0.95 | 0.99 |
| AIC = 601.27 (AIC_R_ = 541.47) | | | | |
| AGGRESSIVENESS | | | | |
| Intercept | 1.63 | 1.29 | 1.97 | <0.001 ^***^ |
| Day | 0.16 | -0.04 | 0.37 | 0.11 |
| Size | 0.11 | -0.33 | 0.56 | 0.63 |
| Condition | -0.09 | -0.55 | 0.37 | 0.70 |
| AIC = 609.51 (AIC_R_ = 599.38) | | | | |
